# Associations of Adiposity and Diet Quality with Serum Ceramides in Middle-Aged Adults with Cardiovascular Risk Factors

**DOI:** 10.3390/jcm8040527

**Published:** 2019-04-17

**Authors:** Margaret A. Drazba, Ida Holásková, Nadine R. Sahyoun, Melissa Ventura Marra

**Affiliations:** 1 Division of Animal and Nutritional Sciences, West Virginia University, Morgantown, WV 26506, USA; madrazba@mix.wvu.edu; 2 Office of Statistics, West Virginia University, Davis College of Agriculture, Natural Resources and Design, West Virginia Agriculture and Forestry Experiment Station, Morgantown, WV 26506-6108, USA; iholaskova@mail.wvu.edu; 3 Department of Nutrition and Food Science, University of Maryland, College Park, MD 20742, USA; nsahyoun@umd.edu

**Keywords:** diet quality, ceramides, obesity, cardiovascular risk, healthy eating index

## Abstract

Rates of adverse cardiovascular events have increased among middle-aged adults. Elevated ceramides have been proposed as a risk factor for cardiovascular events. Diet quality and weight status are inversely associated with several traditional risk factors; however, the relationship to ceramides is less clear. This study aimed to determine associations of adiposity and diet quality with circulating ceramides in middle-aged adults (*n* = 96). Diet quality was estimated using the Healthy Eating Index 2015 (HEI-2015). Serum ceramide concentrations were determined by liquid chromatography–mass spectrometry. A ceramide risk score was determined based on ceramides C16:0, C18:0, and C24:1 and their ratios to C24:0. Participants who were classified as at ‘moderate risk’ compared to ‘lower-risk’ based on a ceramide risk score had significantly higher body mass index (BMI) values, as well as higher rates of elevated fibrinogen levels, metabolic syndrome, and former smoking status. BMI was positively associated with the ceramide C18:0 (R^2^ = 0.31, *p* < 0.0001), the ratio between C18:0/C24:0 ceramides (R^2^ = 0.30, *p* < 0.0001), and the ceramide risk score (R^2^ = 0.11, *p* < 0.009). Total HEI-2015 scores (R^2^ = 0.42, *p* = 0.02), higher intakes of vegetables (R^2^ = 0.44, *p* = 0.02) and whole grains (R^2^ = 0.43, *p* = 0.03), and lower intakes of saturated fats (R^2^ = 0.43, *p* = 0.04) and added sugar (R^2^ = 0.44, *p* = 0.01) were associated with lower C22:0 values. These findings suggest that circulating ceramides are more strongly related to adiposity than overall diet quality. Studies are needed to determine if improvements in weight status result in lower ceramides and ceramide risk scores.

## 1. Introduction

Cardiovascular disease (CVD) and major CVD events such as myocardial infarction (MI) and stroke are largely preventable, yet they remain leading causes of death, disability, and health care spending in the United States (U.S.) [1]. In 2016, a third of all MIs and strokes in the U.S. occurred in 35 to 64-year-old adults [1]. As a result, CVD mortality rates are increasing in middle-aged adults despite declines in the general population over the past few decades [2]. Prevention efforts focus on identifying and treating modifiable risk factors such as dyslipidemia, diabetes, hypertension, and obesity [2]. However, traditional risk factors are not always strong predictors of events. Many patients hospitalized for an MI or stroke have low-density lipoprotein (LDL) levels within a normal range [3], suggesting that those at CVD risk are not being identified before the disease progresses to an event. There is emerging evidence that a class of lipids—ceramides—may play an important role in the pathogenesis of CVD, and may better predict CVD events than some traditional risk factors [4,5,6]. 

Ceramides are a bioactive class of lipids that are synthesized via several molecular pathways, of which the most characterized is de novo synthesis. De novo synthesis begins with palmitoyl CoA and the amino acid l-serine. Then, the resulting sphingoid base is attached to fatty acid side chains of different lengths and degrees of unsaturation, leading to a group of molecules [7] that are diverse in structure and function [8,9]. Ceramides play a role in cell membrane integrity, inflammation, and apoptosis [10], and when they accumulate, they are thought to contribute to the progression of chronic disease, including atherosclerosis [11]. Their low levels in biological samples, large biodiversity, and polar nature made it difficult to quantify ceramides [7]. However, recent advances in mass spectroscopy now allow for the quantification of these small-molecule metabolites, some of which may be prognostic markers for CVD [12]. 

Elevated circulating concentrations of three specific ceramides: N-palmitoyl-sphingosine (C16:0), N-stearoyl-sphingosine (C18:0), and N-nervonoyl-sphingosine (C24:1) were found to be predictive of CVD events in patients with coronary artery disease independent of traditional risk factors [4]. The predictive value was found to be greater when the ceramides were normalized to N-lignoceroyl-sphingosine (C24:0), which is a ceramide that is abundant in the circulation but thought not to be related to CVD [4]. In 2016, the Mayo Clinic began offering a diagnostic test to measure ceramides and assign the risk of a future CVD event based on a ceramide risk score. The risk score is based on six values: C16:0, C18:0, and C24:1, and the ratio of each to C24:0 [5]. Participants classified at higher risk based on their ceramide risk score were four times more likely to suffer a CVD event than those at lower risk [4] independent of age, sex, smoking status, and LDL cholesterol [4,5]. 

Evidence-based clinical care guidelines for reducing elevated ceramide levels and the risk score are not available [13]. It has been suggested that changes in diet may help lower ceramides, and thus CVD risk; however, controlled studies have not been conducted in humans. Higher diet quality and adherence to healthy dietary patterns have been associated with lower CVD risk and mortality [14]. However, no studies have assessed the relationship of diet quality or adherence to United States (U.S.) Dietary Guidelines for Americans (DGAs) as measured by the Healthy Eating Index 2015 (HEI-2015) on circulating ceramides or the ceramide risk score. It is plausible that interventions effective in reducing traditional risk factors also modify circulating ceramides. Studies have shown that circulating ceramides can be modified through exercise [15], statin use [16,17], and weight loss post-bariatric surgery [18,19]. Short-term intervention studies in small samples of healthy humans have shown that caloric excess and increased saturated fat intake increase ceramide levels [20,21,22]. However, there were no significant changes in ceramides after one year in participants at high CVD risk in the PREDIMED (Prevention with Mediterranean Diet) study [23]. 

The relationship between adiposity, diet quality, and ceramides that make up the ceramide risk score is largely unknown. In the U.S., people living in the state of West Virginia experience higher rates of obesity, type 2 diabetes, and hypertension [24], and lower rates of adequate fruit and vegetable intake [25] than any other state in the nation. CVD risk reduction is a public health priority in the state. Middle-aged adults represent a priority population in national health campaigns. The purpose of this study was to determine if diet quality (e.g., adherence to the U.S DGAs), and adiposity were associated with serum ceramides and the ceramide risk score in middle-aged West Virginians with at least one risk factor for CVD. 

## 2. Materials and Methods

### 2.1. Study Design and Sample

In this cross-sectional study, data were analyzed from 96 middle-aged adults (45 to 64 years old) who took part in a larger diet and cardiovascular risk assessment study. Participants were recruited from two counties in north-central West Virginia by word-of-mouth and community advertising. Exclusion criteria included current smokers; diagnosis of cancer or kidney, heart, or liver disease; surgery six months prior; and anti-inflammatory or anticoagulant medications. 

The study consisted of three modes of data collection: multiple telephone interviews to assess dietary intake; an online survey administered using REDCap (Research Electronic Data Capture), which is a secure web-based application designed to support data capture for research studies [26]; and an in-person health assessment. At the in-person health assessment, anthropometric and blood pressure measurements were taken by research staff, and a trained phlebotomist performed a fasting venous blood draw. The study protocol was approved by the West Virginia University (WVU) Institutional Review Board. All the participants provided informed consent before participation and received a $100 gift card upon completion of the study.

### 2.2. Demographic and Health-Related Data

Demographic data and smoking history were self-reported via the online survey. Participants also provided health and medication history at the in-person visit. Blood collected was analyzed by WVU Hospital lab for LDL cholesterol, high-density lipoprotein (HDL) cholesterol, non-HDL cholesterol, triglycerides, glucose, insulin, C-reactive protein (CRP), and fibrinogen. Insulin sensitivity was calculated using the homeostatic model assessment of insulin resistance (HOMA-IR) as fasting insulin (mU L^−1^) × fasting glucose (mmol L^−1^)/22.5 [27] using measured fasting glucose and insulin values. Participants were classified as having a health condition if at least one of the following criteria was met: (1) they reported being diagnosed by a health care provider, (2) they reported taking a medication that is used to treat the condition, or (3) the measured laboratory values or blood pressure (BP) met standard diagnostic cut-off values. The cut-off values for the diagnoses were as follows: pre-diabetes or diabetes, fasting plasma glucose >100 mg/dL [28]; dyslipidemia, LDL ≥100 mg/dL or triglycerides ≥150 mg/dL; hypertension, systolic BP >120 or diastolic BP >80 mm Hg [29]; and a diagnoses of metabolic syndrome required meeting three of the 5 factors defined by the National Cholesterol Education Program Adult Treatment Panel III [30]. 

Blood pressure was measured using the Omron HEM-907XL Intellisense^®^ Automatic Oscillatory Digital Blood Pressure monitor (Omron Health Care, Lake Forest, IL, USA) [31]. A single assessor performed all the blood pressure measurements. Arm circumference was measured to the nearest 0.1 cm to determine appropriate cuff size based on manufacturer recommendations. With the participant in a seated upright position and after an initial rest of 5 min, the machine took three blood pressure measurements at 30-second intervals; the average reading was used for analysis [32]. Anthropometric and body composition measurements were taken using standardized protocols, with participants fasted, lightly clothed, and without shoes. Measurements were recorded in duplicate, and averages were used for analysis. Height (cm) was measured using the Seca 274 digital mobile stadiometer (Seca, Hamburg, Germany). Weight (kg) and fat mass index (FMI) (kg/m^2^) were measured using the Seca medical Bioelectrical Composition Analyzer (mBCA) 514 (Seca, Hamburg, Germany). Body mass index (BMI) was calculated as weight (kg)/height (m^2^) and was classified using World Health Organization classifications [33]. Waist and hip circumferences (cm) were measured using a Gulick II Tape Measure. Waist circumference (WC) was measured at the iliac crest; values >102 cm for men and >88 cm for women were classified ‘at risk’ [33]. Hip circumference was measured at the maximum point of protuberance of the buttocks. Waist–hip ratio (WHR) was calculated as waist circumference (cm)/hip circumference (cm); values were classified as ‘at risk’ if WHR was ≥0.90 cm for men and ≥0.85 cm for women [33].

### 2.3. Diet Quality Assessment

Dietary intake data were collected and analyzed using three 24-hour dietary recalls and Nutrition Data Systems for Research (NDSR) software version 15 (2015) developed by the Nutrition Coordinating Center, University of Minnesota, Minneapolis, MN. Self-reported dietary intake was obtained via telephone interview by trained research personnel using the NDSR four-pass method on non-consecutive days (one weekend and two weekdays). Food and nutrient data was transformed into the HEI-2015 metric, which measures adherence to the 2015–2020 Dietary Guidelines for Americans (DGAs) [34] based on 13 components (nine adequacies and four moderation). To calculate the HEI-2015 scores, NDSR output data was transformed into HEI component variables using NDSR’s unpublished guide [35]. Food group servings (total fruits, whole fruits, total vegetables, greens and beans, whole grains, dairy, total protein foods, seafood and plant proteins, and refined grains) were converted to total servings per 1000 kilocalories and sodium intake was converted to mg per 1000 kilocalories. A ratio of polyunsaturated fatty acids (PUFAs) and monounsaturated fatty acids (MUFAs) to saturated fatty acids (SFAs) was generated by dividing the sum of PUFAs and MUFAs by SFAs. Added sugars and saturated fats were assessed as percent of kilocalories. For the four moderation components—refined grains, sodium, added sugars, and saturated fats—higher scores represent lower intakes. Once the NDSR components were in units consistent with the HEI metric, the simple HEI scoring algorithm method for multiple days of intake data was applied [36]. Component scores were summed for a total score ranging from 0–100, with higher scores indicating better adherence to the 2015–2020 U.S. DGAs.

### 2.4. Ceramides Analysis

Lipidomics analysis was conducted by the WVU Metabolomics Core using liquid chromatography–mass spectrometry (LC-MS). Serum samples were extracted using a modified Bligh and Dyer procedure using C12:0-ceramide as an internal standard (Avanti Polar Lipids, Alabaster, AL, USA) using previously described methods [37,38]. Following liquid–liquid extraction, the organic layer was dried under nitrogen gas (Organomation Associates Inc., Berlin, MA, USA) and resuspended in pure methanol before analysis. Ceramides were separated by gradient elution using ultra high-pressure liquid chromatography (ExionLC AD, SCIEX, Framingham, MA, USA) on a C18 reverse-phase column (Phenomenex, Torrence, CA, USA). Ceramides were detected using electrospray ionization tandem mass spectrometry (ESI-MS/MS) as previously described (QTRAP 5500, SCIEX) [39]. Ionspray source voltage was 5000 V at a temperature of 500 °C. Nebulizer, heater, curtain, and collision gas pressures were maintained at 70, 60, 28, and 9 psi, respectively. Ceramide ionization parameters were optimized individually, ranging from a declustering potential of 30 to 50 V, an entrance potential of 10 to 15 V, collision energy of 32 to 37 V, and a collision cell exit potential of 13 to 17 V. Ceramides were measured by multiple reaction monitoring of the protonated molecular ion with a transition ion of 264.2 m/z. Eleven-point calibration curves (0.1 ng/mL to 10 µg/mL) were constructed by plotting the area under the curve for C16:0-ceramide, C18:0-ceramide, C22:0-ceramide, and C24:0-ceramide (Avanti Polar Lipids). Fluctuations in extraction and ionization efficiencies were controlled by normalizing to the C12:0-ceramide response, and samples were re-run if the internal standard response deviated more than 20% from its median value. Concentrations were determined by curve fitting the identified ceramide species based on acyl-chain length. Standards were injected in duplicate to ensure similar response (the overall mean CV was 12.08%; R^2^ ≥ 0.985). Instrument control and quantitation were performed using Analyst 1.6.3 and MultiQuant 3.0.2 software, respectively (SCIEX). 

### 2.5. Ceramides Risk Score

The ceramides used in this analysis included the six variables that make up the ceramide risk score (C16:0, C18:0, C24:1, C16:0/C24:0, C18:0/C24:0, and C24:1/C24:0) [40] and two additional ceramides (C20:0 and C22:0) that were identified in the literature as being related to diet [20,41], adiposity [42], or CVD risk [15,23,43]. Ceramides (ng/mL) were converted to units that were consistent with the ceramide risk score (µmol/L). Ceramide risk scores were calculated by assigning a value between 0–2 to each of the six risk score components based on published cut-off values [4,5]. The six component scores were summed for a total ceramide risk score ranging from 0 to 12, with higher scores indicating a higher risk of adverse cardiovascular events. The scores were categorized into risk groups: lower risk (0–2), moderate risk (3–6), and increased risk (7–9) [4]. No participant scores met a fourth category: higher risk (10–12). For analysis purposes in this study, the ‘increased risk’ category was combined into the ‘moderate risk’, as only three participants had scores in the increased risk category.

### 2.6. Statistical Analysis

Demographic and health-related data were reported as means and standard errors of the mean (SEM) or frequency with percentage, when appropriate. Ceramides and risk factors for CVD (i.e., cholesterol, triglycerides, glucose) were log-transformed to achieve a normal distribution for analysis. Medians and interquartile ranges were reported for non-normally distributed variables. To assess differences between ceramide risk score categories for all the demographic and clinical characteristics, Student’s *t*-test or Chi-square tests were performed when appropriate. Bivariate analysis was performed to assess the relationship between potential confounding variables (demographics, health conditions, CVD biomarkers, inflammatory markers, and lifestyle factors) and each ceramide and the ceramide risk score. The Benjamini–Hochberg procedure was performed to control for excessive Type I error due to multiple analyses, with a false discovery rate set to 10%. The variables that remained significant after the Benjamini–Hochberg procedure were entered into a stepwise regression model, with alpha-to-enter set at 0.15 and alpha-to-remove set at 0.15. This enabled us to estimate the effect of multiple variables on ceramides and the ceramide risk score. The variables that remained significant were included in the adjusted model. Multiple linear regression models were done to assess the hypothesized relationship between the HEI-2015 diet quality score and BMI on a continuous scale with individual ceramides along with the ceramide risk score. These models enabled us to assess if the HEI-2015 or BMI were associated with ceramide levels when adjusted for other significant clinical, demographic, or lifestyle characteristics. The 13 HEI-2015 components were analyzed further using multiple linear regression models with individual ceramides and the ceramide risk score. BMI was further analyzed categorically against each individual ceramide and the ceramide risk score using one-way ANOVA and Tukey’s HSD (honestly significant difference) test for comparison of BMI categories (normal, overweight, and obese).

All data analyses were performed using JMP and SAS software (JMP^®^, Version Pro 12.2, SAS Institute Inc., Cary, NC, USA, Copyright ©2015; SAS^®^, Version 9.4, SAS Institute Inc., Cary, NC, USA, Copyright ©2002–2012). Power of the test was determined as 80% for the relationship of BMI and the ceramide risk score. Significance criterion alpha for all tests was 0.05.

## 3. Results

### 3.1. Participant Characteristics by Ceramide Risk Category

Overall, most participants were non-Hispanic white (95.8%), college educated (57.3%), and had annual household incomes >$50,000 (67.4%). Demographic and health-related characteristics by ceramide risk category are presented in Table 1. The mean age of the sample was 54 ± 4.7 years old Over half were women (57.3%). The mean BMI was 30.85 ± 7.23; 51.1% were classified as obese. All the participants had at least one cardiovascular risk factor; 92% had dyslipidemia (controlled and uncontrolled). Participants classified at ‘moderate risk’ compared to ‘low-risk’ based on ceramide risk category had significantly higher BMI (*p* = 0.003), WC (*p* = 0.005), and FMI (*p* = 0.004), elevated fibrinogen levels (35.0% versus 14.3%, *p* = 0.02), higher rates of metabolic syndrome (52.5% versus 28.6%, *p* = 0.02), and had formerly smoked (62.5% versus 1.1%, *p* = 0.04).

The most abundant circulating ceramides were C24:0 followed by C22:0 and C24:1. Ceramide risk scores ranged from 0 to 8 out of a possible 12 points; 58.3% were in the low-risk group, and 41.7% were in the moderate risk or increased risk groups. Supplemental Table S1 shows quantities of ceramides by risk category.

### 3.2. Relationship between Potential Covariates and Ceramide

Age, gender, income, statin use, and hypertension were not significantly associated with any of the ceramides or the risk score. All of the adiposity measures (i.e., WC, WHR, and FMI); several laboratory values (LDL, HDL, non-HDL, triglycerides, glucose, insulin, HOMA-IR, fibrinogen, and CRP); having a diagnosis of diabetes or metabolic syndrome, and being a former smoker were each significantly associated with at least one ceramide or the risk score. However, after stepwise regression, only LDL, HDL, non-HDL, glucose, FMI, fibrinogen, and smoking status remained significantly associated with individual ceramides or the risk score, and therefore were the only covariates included in the adjusted models. Supplemental Table S2 depicts the results of bivariate analyses of associations between traditional CVD risk factors and ceramides.

### 3.3. Relationship of BMI (Adiposity) and HEI (Diet Quality) with Ceramides

Table 2 shows the associations between BMI and HEI scores with ceramides. BMI was associated with C18:0, C16:0/24:0, C18:0/C24:0, and the ceramide risk score in unadjusted models, and remained positively associated with C18:0, C18:0/C24:0, and the ceramide risk score after adjusting for covariates. The diet quality scores measured by HEI-2015 ranged from 25.6 to 87.7 out of 100 points. The mean score was 54.05 ± 1.45, indicating ‘needing improvement’ (HEI scores 51–80). Diet quality was inversely associated with only one ceramide, C22:0, in both unadjusted and adjusted models. It was not associated with any of the ceramides that make up the risk score. Ceramides C16:0, C24:0, and C24:1/24:0 were not associated with any of the outcome variables. 

Table 3 depicts the associations between individual HEI-2015 component scores and ceramides. After adjusting for confounding risk factors, HEI scores that represent the recommended intakes for total vegetables, whole grains, saturated fats, and added sugars were each inversely associated with C22:0. That is, higher intakes of vegetables and whole grains and lower intakes of saturated fats and added sugar were associated with lower C22:0 values. Absolute saturated fat intake (grams per day) was significantly and positively associated with C22:0 (R^2^ = 0.41, *p* = 0.04). Individual HEI components (i.e., whole fruit, total fruit, greens and beans, dairy, total protein foods, seafood and plant protein, unsaturated to saturated fatty acid ratio, and sodium) were not associated with any ceramides or the ceramide risk score. 

**Figure 1:**
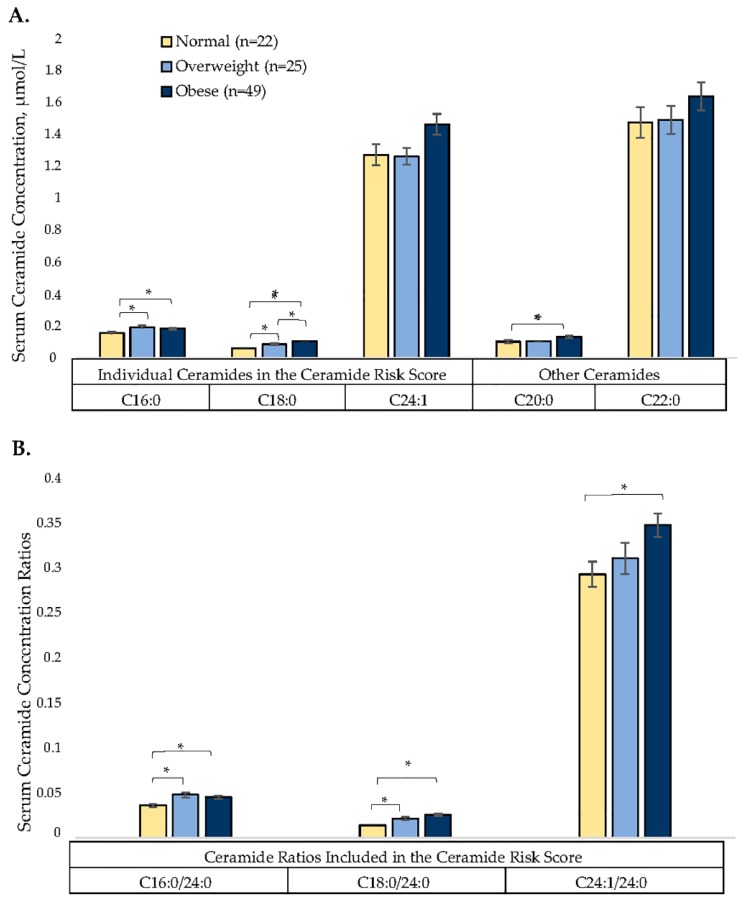
(**A**) Serum ceramide concentrations (µmol/L) by BMI category: normal weight (18.5–24.9 kg/m^2^), overweight (25–29.9 kg/m^2^), and obese (≥30 kg/m^2^). Values reported are mean concentrations. (**B**) Ceramide ratios included in the risk score by BMI category. Values are means of ratios. Tukey’s honestly significant difference (HSD) was used to test significance between the three BMI categories. * Significant *p*-values < 0.05.

**Figure 2:**
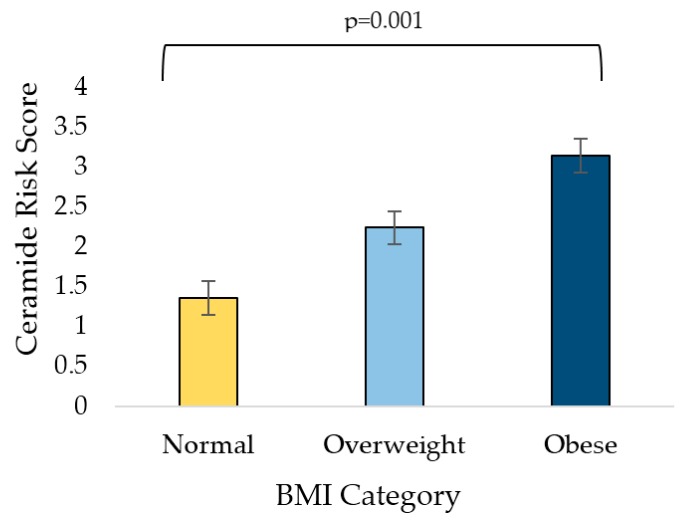
Ceramide risk score by BMI category: normal (18.5–24.9 kg/m^2^), overweight (25–29.9 kg/m^2^), and obese (≥30 kg/m^2^). Values represent means and SEM. Tukey’s HSD was used to test significance between categories.

Figure 1 depicts ceramide concentrations by BMI category. Of the 96 participants, 22.9% were classified as normal weight, 26.0% were classified as overweight, and 51.0% were classified as obese. Five of the six values that make up the ceramide risk score (C16:0, C18:0, C16:0/24:0, C18:0/24:0, C24:1/24:0, and C20:0) were all significantly higher in participants classified as obese compared to normal weight. Four of the six values in the risk score (C16:0, C18:0, C16:0/24:0, and C18:0/24:0) were significantly higher in participants classified as overweight compared to normal weight. Figure 2 shows the ceramide risk score by BMI category. The risk score increased by BMI category and was significantly higher between participants classified as normal weight versus obese (1.36 ± 0.24 versus 3.14 ± 0.31, *p* = 0.001).

## 4. Discussion

This is the first study to examine the cross-sectional associations of adiposity and diet quality with the ceramide risk score, which is an emerging risk factor for CVD. We detected that even between the two lowest ceramide risk score categories, there were significant differences between groups for adiposity (BMI and FMI), and rates of elevated fibrinogen (a proinflammatory mediator), metabolic syndrome, and former smoker status. BMI was positively associated with the ceramide risk score and two of the six ceramide values in the risk score (C18:0 and C18:0/24:0). Total HEI-2015 scores and four diet component scores (total vegetables, whole grains, saturated fats, and added sugars) were inversely associated with only one ceramide, C22:0, which is a ceramide associated in the literature with diet [20,41] but not included in the ceramide risk score. Higher diet component scores indicate improvements in diet quality. That is, higher scores represent higher intakes of vegetables and whole grains, but lower intakes of saturated fats and added sugar. 

Similar to other studies, the most abundant serum ceramides were C24:0 and C24:1 [44,45]. Ceramide C24:0 was positively associated with LDL, non-HDL, and triglycerides, as has been reported in other studies [4], but inversely associated with fibrinogen in our population. We did not see associations between statin use and ceramides. Study results have been inconsistent, with some studies showing lower circulating ceramides in participants taking statins [42,46] and others such as ours, did not [42]. In our population, 91.7% of participants had dyslipidemia. However, 24% reported statin use, but of those, 60.9% still had high lipid levels, suggesting that the statins were ineffective or that participants were not regularly taking their medications. Additionally, significantly more participants with metabolic syndrome were in the moderate ceramide risk category compared to the low-risk category. This finding is consistent with other studies that ceramides may be inducers of metabolic disorders [47].

### 4.1. Associations between Adiposity and Ceramides

BMI was positively associated with the ceramide risk score, C18:0, and C18:0/24:0 after adjusting for confounding variables. To our knowledge, no other studies have compared BMI with this specific total ceramide risk score, but studies have reported an association between BMI and various circulating ceramide species. Circulating C18:0 was associated with BMI in several studies [4,42,44]. Consistent with our study, Meeusen et al. reported an association between BMI and two of the six risk components of the risk score (C18:0 and C18:0/C24:0); however, the association was minimal (R^2^ value <3%) compared to the 30% in this study [4]. Differences in populations and overall sample size (*n* = 495 versus *n* = 96) may account for variation in the strength of the associations. In contrast, Mielke et al. reported additional associations of BMI with C16:0, C24:1, C20:0, and C22:0 among middle-aged subjects with an average BMI of 26 kg/m^2^ (overweight) [42] that we did not detect. In a small sample, Haus et al. detected higher concentrations of C18:0, C20:0, and C24:1 ceramides in subjects with type 2 diabetes and obesity (*n* = 13) compared to lean healthy control subjects (*n* = 14) [44]. A possible mechanism for increased serum ceramides is that in obese humans, circulating free fatty acids, which serve as substrates for ceramide synthesis via the de novo pathway, are elevated, leading to an overproduction of ceramides [48,49]. Ceramides can be synthesized in several different tissues, but those synthesized in the liver can be readily incorporated in very low-density lipoprotein (VLDL) particles and released into the circulation [50]. Ceramides circulate in the blood mainly associated with lipoprotein particles, VLDL and LDL [50]. Thus, overproduction in the liver is a potential source for elevated circulating ceramides. Excess ceramides are postulated to be the mechanistic link between obesity and obesity-related chronic conditions. Thus, our research adds to literature suggesting a link between obesity and a potential overproduction and accumulation of C18:0 species and the overall ceramide risk score. Studies are needed that determine if dietary interventions for weight loss result in a reduction of the ceramides in the risk score. 

### 4.2. Associations between Diet Quality and Ceramides

We did not detect associations between diet quality and ceramide species in individual ceramides in the risk score or total risk score; however, greater HEI scores were associated with lower C22:0 concentrations after adjusting for LDL and non-HDL cholesterol. We found that 42% of the variation in C22:0—a very-long-chain ceramide with a saturated acyl chain—was explained by the HEI scores indicating a strong association. There is limited research on the associations between diet and ceramides. This was the first study to assess the relationship between the a priori HEI metric of diet quality and ceramides as a marker for CVD risk. However, other studies have shown that the HEI score was related to reductions in other CVD risk factors such as LDL [51] and reductions in CVD mortality [14,52]. 

While C22:0 is not part of the ceramide risk score, previous studies showed that it is positively associated with CVD risk in humans [23,43]. Two intervention studies that measured C22:0 found that it was modifiable by diet [20,41]. One intervention of 200 subjects who were between 30–65 years old with metabolic syndrome aimed to determine how a Nordic diet affected the lipidomic profile compared to control subjects. Subjects in the intervention consumed a dietary pattern of higher amounts of fiber, vegetables, fruits, berries, and fish, lower intakes of salt and sugar, and a higher quality of dietary fat than the control for 18 to 24 weeks. The control group received low-fiber cereal products and dairy fat such as butter [41]. Results indicated a significant decrease in C22:0, C23:0, and C24:0 concentrations compared to the control group after 12 weeks, but not after 24 weeks [41]. In another study, Heilbronn et al. also observed an elevation in C22:0 by 25% in subjects overfed by 1250 kilocalories per day for 28 days [20]. 

When assessing individual HEI components, we found that participants had higher scores, indicating that better adherence to recommendations for saturated fat intake (≤8% of energy intake) had lower C22:0 concentrations. Additionally, participants who consumed higher amounts of absolute saturated fat (grams per day) had higher C22:0. De novo ceramide synthesis depends on the availability of free fatty acids, specifically the saturated fat palmitate [10]. The overconsumption of this saturated fat may result in excess ceramide accumulation [10]. Additionally, in our population, better adherence to the DGAs for total vegetable intake, whole grain intake, and added sugar intake was also associated with lower C22:0. This suggests that multiple components of the diet work synergistically, and that dietary patterns should be studied in relationship to ceramides in larger samples. Future studies on the relationship between ceramides and diet quality are needed, specifically long-term intervention studies to assess changes in ceramides after improvements in diet quality.

### 4.3. Limitations

There are several limitations to this study. First, our sample size was small and used a convenience sample whose ceramide risk scores fell largely within the two lowest groups of risk. A larger sample may have included a broader range of risk scores, and associations may have been detected when the lowest versus highest scores are compared. Second, the majority of participants were non-Hispanic white, which is representative of the population in West Virginia, but makes it difficult to generalize findings to other populations. Third, the cross-sectional study design does not allow for cause–effect relationships. Studies are needed to investigate long-term changes in ceramides over time. Lastly, the study used self-reported data, such as dietary intake, medications, and medical conditions. There are inherent limitations with self-reported intake data, specifically the under-reporting of dietary intake or failing to report all the medications used.

### 4.4. Clinical implications

Laboratory tests are currently available that quantify ceramides and categorize risk of CVD events.. However, studies have not yet been conducted to inform evidence-based guidelines on treatments to effectively modify ceramides should they be elevated. It is plausible that diet, lifestyle, and lipid-lowering medications known to reduce traditional risk factors may also be effective in lowering ceramides. Large epidemiological studies to confirm the relationship between specific ceramides and CVD events, and interventional studies to determine the effectiveness of treatment strategies in reducing ceramides and CVD events are urgently needed. Until this data is available, it seems premature to use ceramides as a therapeutic target in clinical practice. 

## 5. Conclusions

This study showed that middle-aged adults with obesity had higher circulating C18:0 and C18:0/24:0, and higher ceramide risk scores than those who were normal weight or overweight. Higher BMI remained independently associated with higher ceramide levels after adjusting for confounding variables. Future studies are needed to determine if a reduction in weight status results in lower ceramide risk scores in humans, and if interventions to improve diet quality would be effective in lowering ceramides and the ceramide risk score. 

## Figures and Tables

**Table 1:** Participant characteristics by ceramide risk category.

	All *N* = 96	Lower Risk ^1^ *n* = 56	Moderate Risk ^2^ *n* = 40	*p*-Value ^3^
**Demographic Factors**				
Age, year	54.30 ± 0.47	53.95 ± 0.55	54.8 ± 0.84	0.40
Sex, women	55 (57.3%)	33 (58.9%)	22 (55.0%)	0.70
**Health-Related Factors**				
Adiposity				
Body Mass Index, kg/m^2^	30.85 ± 0.74	29.22 ± 0.92	33.13 ± 1.13	0.003
Waist Circumference, cm	103.35 ± 1.67	99.41 ± 2.07	108.87 ± 2.55	0.005
Elevated Waist Circumference ^4^	70 (72.9%)	37 (66.1%)	33 (82.5%)	0.07
Waist-to-Hip Ratio, cm	0.90 ± 0.008	0.89 ± 0.01	0.91 ± 0.01	0.16
Elevated Waist-to-Hip Ratio ^5^	63 (65.6%)	36 (64.3%)	27 (67.5%)	0.74
Fat Mass Index, kg/m^2^	12.26 ± 0.55	11.00 ± 0.65	14.02 ± 0.89	0.004
**Laboratory Values**				
Total Cholesterol ≥200 mg/dL	52 (54.2%)	30 (53.6%)	22 (55.0%)	0.89
LDL ≥100 mg/dL	77 (81.1%)	43 (76.8%)	34 (87.2%)	0.20
Low HDL ^6^	22 (22.9%)	12 (21.4%)	10 (25.0%)	0.68
Triglycerides ≥150 mg/dL	18 (18.8%)	9 (16.1%)	9 (22.5%)	0.43
Glucose >100 mg/dL	35 (36.5%)	19 (33.9%)	16 (40.0%)	0.54
Insulin >24 mg/dL	4 (4.17%)	1 (1.8%)	3 (7.5%)	0.17
HOMA-IR	2.18 ± (0.17)	1.97 (0.19)	2.48 (0.31)	0.19
CRP ≥8 mg/dL	16 (16.7%)	9 (16.1%)	7 (17.5%)	0.85
Fibrinogen >400 mg/dL	22 (22.9%)	8 (14.3%)	14 (35.0%)	0.02
**Medical Conditions**				
Metabolic Syndrome	37 (38.5%)	16 (28.6%)	21 (52.5%)	0.02
Diabetes	39 (40.6%)	22 (39.3%)	17 (42.5%)	0.75
Dyslipidemia	88 (91.7%)	50 (89.3%)	38 (95.0%)	0.32
Hypertension	37 (38.5%)	18 (32.1%)	19 (47.5%)	0.13
Statin use	23 (24.0%)	13 (23.2%)	10 (25.0%)	0.84
**Lifestyle Factors**				
Former Smoker	48 (50.0%)	23 (41.1%)	25 (62.5%)	0.04
HEI-2015 Diet Scores	54.05 ± 1.45	55.27 ± 1.73	52.36 ± 2.49	0.34

Values are means ± SEM for continuous variables or *n* (%) for categorical variables. Abbreviations: LDL, low-density lipoprotein; HDL, high-density lipoprotein; HOMA-IR, homeostatic model assessment of insulin resistance; CRP, C-reactive protein; HEI-2015, Healthy Eating Index 2015. ^1^ Lower risk: ceramide risk score 0–2. ^2^ Moderate risk: ceramide risk score 3–6, and three participants with scores of 7 (*n* = 2) and 8 (*n* = 1). ^3^ Student’s *t*-test or chi-square tests were used to test significance; significant *p*-value <0.05. ^4^ Elevated waist circumference is >102 cm for men and >88 cm for women. ^5^ Elevated waist-to-hip ratio is ≥0.90 cm for men and ≥0.85 cm for women. ^6^ Low HDL is <40 mg/dL for men and <50 mg/dL for women.

**Table 2:** Associations between BMI (adiposity) and HEI-2015 (diet quality) with ceramides.

Outcome Variables	Ceramides Included in the Ceramide Risk Score				Ceramide Risk Score	Other Ceramides	
	C18:0	C24:1	C16:0/24:0	C18:0/24:0		C20:0	C22:0
**Unadjusted Model**							
BMI (kg/m^2^)	0.80; 0.19 (<0.001)	NS	0.36; 0.07 (0.006)	1.02; 0.26 (<0.001)	5.97; 0.07 (0.006)	NS	NS
HEI-2015	NS	NS	NS	NS	NS	NS	−0.002; 0.06 (0.009)
**Adjusted Models**							
BMI (kg/m^2^)	0.81; 0.31 (<0.0001)	NS	NS	0.91; 0.30 (<0.0001)	5.58; 0.11 (0.009)	NS	NS
HEI-2015	NS	NS	NS	NS	NS	NS	−0.002; 0.42 (0.02)

Values reported are slope per unit change; coefficient of determination R^2^ and (significant *p*-value). Abbreviations: HEI-2015, Healthy Eating Index 2015; BMI, body mass index; NS, not significant. Covariates used in adjusted models varied by ceramide: C18:0 (HDL-C, glucose); C24:1 (LDL, non-HDL); C16:0/24:0 (glucose, fibrinogen); C18:0/24:0 (glucose); C24:1/24:0 (smoking); ceramide risk score (smoking); C20:0 (LDL, non-HDL); C22:0 (LDL, non-HDL).

**Table 3:** Associations between the Healthy Eating Index (HEI-2015) components and ceramides.

HEI Component	Ceramides in Risk Score			Other Ceramides	
	C16:0	C24:1	C16:0/24:0	C20:0	C22:0
Unadjusted Models					
Total Vegetables	−0.02; 0.04 (0.02)	NS	NS	NS	−0.03; 0.05 (0.01)
Whole Grains	NS	NS	NS	−0.01; 0.04 (0.03)	−0.01; 0.04 (0.03)
Refined Grains	NS	NS	−0.007; 0.04 (0.03)	NS	NS
Saturated Fats	NS	−0.01; 0.05 (0.01)	NS	−0.02; 0.06 (0.01)	−0.02; 0.08 (0.003)
Added Sugar	NS	NS	NS	NS	−0.01; 0.07 (0.007)
Adjusted Models					
Total Vegetables	NS	NS	NS	NS	−0.02; 0.44 (0.02)
Whole Grains	NS	NS	NS	NS	−0.007; 0.43 (0.03)
Saturated Fats	NS	NS	NS	NS	−0.008; 0.43 (0.03)
Added Sugar	NS	NS	NS	NS	−0.009; 0.44 (0.01)

Values reported are slope per unit change; coefficient of determination R^2^ and (p-value). Abbreviations: HEI-2015, Healthy Eating Index 2015; NS, not significant. Ceramides not significant with any HEI-2015 components were C18:0, C24:0, C18:0/24:0, C24:1/C24:0 or the ceramide risk score (not shown). Covariates used in adjusted models varied by ceramide: C16:0 (HDL, non-HDL, glucose); C24:1 (LDL, non-HDL); C16:0/24:0 (glucose, fibrinogen); C20:0 (LDL, non-HDL); C22:0 (LDL, non-HDL). Significant *p*-value <0.05.

## Supplementary Materials

The following are available online at https://www.mdpi.com/2077-0383/8/4/527/s1, Table S1: Ceramides concentrations and ceramide risk scores (CRS) by risk category; Table S2: Bivariate analysis of ceramides by risk factors for cardiovascular disease to assess for possible covariates.
