# Supplementary material for: Associations of Adiposity and Diet Quality with Serum Ceramides in Middle-Aged Adults with Cardiovascular Risk Factors
Source: J Clin Med. 2019 Apr 17;8(4):527. doi: 10.3390/jcm8040527 (PMC6517875; doi:10.3390/jcm8040527)
Supplement: Supplementary file 1 [file jcm-08-00527-s001.pdf]

Supplemental Table 1. Ceramides concentrations and ceramide risk scores (CRS) by risk category.

Ceramides, Ratio or CRS	All (n=96)	Lower Risk (n=56)	Moderate Risk (n=40)	p-value
C16:0	0.18 (0.10-0.32)	0.17 (0.10-0.26)	0.20 (0.13-0.33)	<.0001
C18:0	0.09 (0.03-0.19)	0.07 (0.03-0.11)	0.12 (0.06-0.19)	<.0001
C20:0*	0.12 (0.05-0.29)	0.10 (0.05-0.22)	0.14 (0.07-0.29)	<.0001
C22:0*	1.56 (0.67-4.48)	1.49 (0.67-2.63)	1.66 (0.74-4.48)	0.2478
C24:0*	4.32 (2.05-8.81)	4.40 (2.75-6.45)	4.22 (2.05-8.81)	0.1463
C24:1*	1.37 (0.70-2.99)	1.19 (0.70-2.00)	1.62 (0.82-2.99)	<.0001
C16:0/C24:0	0.04 (0.03-0.08)	0.04 (0.03-0.06)	0.05 (0.03-0.08)	<.0001
C18:0/C24:0*	0.02 (0.01-0.05)	0.02 (0.01-0.04)	0.03 (0.02-0.05)	<.0001
C24:1/C24:0	0.33 (0.16-0.63)	0.28 (0.16-0.39)	0.40 (0.22-0.63)	<.0001
Ceramide Risk Score*	2.5 (0-8)	1.05 (0-2)	4.53 (3-8)	

Values represent mean ceramide concentrations μmol/L (range). Independent t-test assuming unequal variance was used to compare ceramide values for 'lower risk' (0-2) vs. 'moderate risk' (3-6) categories unless data was not normally distributed (indicated via *), in which case a nonparametric Wilcoxon test was applied.

Supplemental Table 2. Bivariate analysis of ceramides by risk factors for cardiovascular disease to assess for possible covariates.

Risk Factor	C16:0	C18:0	C24:1	C16:0/24:0	C18:0/24:0	C24:1/24:0	C24:0	Risk Score	C20:0	C22:0
FMI	NS	0.37; 0.14 (<0.001)	NS	0.21; 0.08 (0.003)	0.53; 0.25 (<0.001)	0.17; 0.06 (0.01)	-0.15; 0.06 (0.01)*	2.86; 0.05 (0.01)	NS	NS
LDL	NS	NS	0.0009; 0.05 (0.02)*	NS	NS	NS	0.001; 0.12 (<0.001)*	NS	0.001; 0.04 (0.03)*	0.001; 0.11 (<0.001)*
HDL	-0.35; 0.16 (<0.001)*	-0.68; 0.21 (<0.001)*	NS	-0.24; 0.04 (0.02)	-0.58; 0.12 (<0.001)	NS	NS	NS	-0.35; 0.06 (0.007)	-0.33; 0.07 (0.004)
Non-HDL	0.25; 0.05 (0.01)*	NS	0.47; 0.17 (<0.001)*	-0.30; 0.05 (0.02)	NS	NS	0.55; 0.28 (<0.001)*	NS	0.69; 0.20 (<0.001)*	0.73; 0.30 (<0.001)*
Glucose	0.67; 0.07 (0.008)*	1.34; 0.09; (0.002)*	NS	0.92; 0.08 (0.003)*	1.58; 0.11 (<0.001)*	NS	NS	NS	NS	NS
Fibrinogen	NS	NS	NS	0.45; 0.11 (<0.001)*	0.63; 0.09 (0.002)	0.32; 0.06 (0.01)	-0.43; 0.14 (<0.001)*	NS	NS	NS
Smoking status	NS	NS	NS	NS	NS	0.08 (0.004)*	0.05 (0.02)	0.05 (0.02)*	NS	0.05 (0.02)
WC, cm	NS	0.004; 0.18 (<0.001)	NS	0.002; 0.06 (0.02)	0.005; 0.22 (<0.001)	0.002; 0.05 (0.03)	NS	0.030; 0.06 (0.02)	NS	NS
WHR, cm	NS	0.62; 0.08 (0.006)	NS	NS	NS	NS	NS	NS	NS	NS
Triglycerides	NS	0.40; 0.15 (<0.001)	0.16; 0.07 (0.01)	NS	NS	NS	0.18; 0.10 (0.003)	NS	0.40; 0.22 (<0.001)	NS
Insulin	NS	0.23; 0.19 (<0.001)	NS	0.08; 0.04 (0.04)	0.26; 0.20 (<0.001)	NS	NS	1.44; 0.05 (0.02)	0.15; 0.10 (0.002)	NS
HOMA-IR	0.07; 0.06 (0.02)	0.23; 0.18 (<0.001)	NS	0.10; 0.06 (0.02)	0.25; 0.18 (<0.001)	NS	NS	NS	0.15; 0.10 (0.002)*	NS
CRP	NS	0.12; 0.07 (0.01)	NS	NS	0.15; 0.09 (0.004)	NS	NS	NS	NS	NS
Diabetes	NS	0.06 (0.006)	NS	NS	0.04 (0.03)	NS	NS	NS	NS	NS
MetS	NS	0.06 (0.008)	NS	NS	0.09 (<0.001)	NS	NS	NS	NS	NS

Values reported are slope per unit change; coefficient of determination (R_2) (p-value). For smoking, diabetes, and MetS, R_2 and p-value reported. *p-values significant in bivariate analysis that remained significant after stepwise regression and were included in the adjusted model. Abbreviations: LDL, low density lipoprotein; HDL, high density lipoprotein; WC, waist circumference; WHR, waist-to-hip ratio; HOMA-IR, homeostatic model assessment of insulin resistance; CRP, c-reactive protein; MetS, Metabolic Syndrome; NS, non-significant. Variables not significant with any ceramides include: age, gender, income, statin use, and hypertension. .
